# Investigation of long-term pressure on primary packaging materials and a biologic drug product for injection with a novel autoinjector concept

**DOI:** 10.1007/s13346-024-01612-y

**Published:** 2024-05-10

**Authors:** Daniel Primavessy, Max Piening, Adam Nightingale, Heather Jameson, Matthew Latham, James Alexander, Sarah Büttner, Juergen Pfrang, Andreas Zapf, Tom Oakley, Andreas Brutsche, Sigrid Saaler-Reinhardt

**Affiliations:** 1 Midas Pharma GmbH, Rheinstrasse 49, 55218 Ingelheim, Germany; 2 Springboard Pro Ltd, St. John’s Innovation Centre, Cowley Road, Cambridge, CB4 0WS UK; 3 Gerresheimer Regensburg GmbH, Oskar-von-Miller-Strasse 6, 92442 Wackersdorf, Germany; 4 EDUMO Consulting, Professor-Neeb-Strasse 4a, 55291 Saulheim, Germany; 5 Institut für Organismische und Molekulare Evolutionsbiologie, Johannes Gutenberg-Universität Mainz, Becherweg 32, 55099 Mainz, Germany

**Keywords:** Cartridge, Drug-device combination, Monoclonal antibody, Combination product, Medical device, Drug product

## Abstract

Gerresheimer and Midas Pharma have developed a novel cartridge-based autoinjector concept in which the cartridge as primary packaging is under constant pressure. In this article standard cartridge primary packaging material of five different companies were analyzed for their behavior under long-term pressure. Materials of 3 glass manufacturers and 2 manufacturers for cartridge rubber parts were considered. Within the test program septum stability, septum piercing, glide forces (GF), break-loose forces (BLF), glass breaking as well as a regulatory approved and marketed antibody drug product under pressure were subject to analysis. Under pressure the cartridge septum bulge grew within the first 14 days and then relevantly slowed down. An accelerated study in different atmospheric conditions allowed to extrapolate values for 24 months storage, not showing any signs of decay or problematic septum bulge increase. Pierce forces were in normal ranges and septum rupture could not be observed at the end of 42 days of pressurization. GF and BLF were within acceptable ranges and changes due to pressure could not be observed. Lowest glass breaking pressures at 4922 kPa turned out to be at least 3.5 times higher than pressures used in the autoinjector concept. Degradation of the Adalimumab antibody drug product due to pressure or device fluid pathway could not be observed with size exclusion chromatography, electrophoresis or sub-visible particles tested as a release testing in a GMP setting.

**Graphical Abstract:**

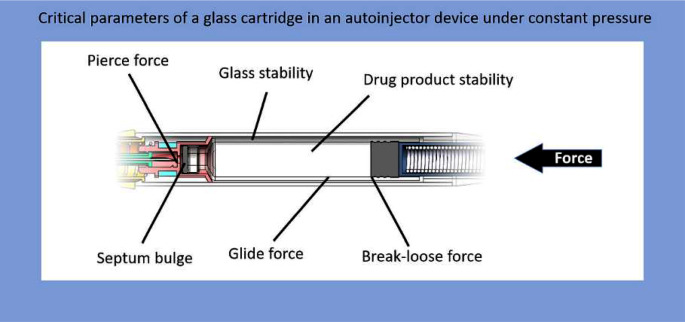

## Introduction

Handheld injection devices for self-administration by patients, or commonly called autoinjectors have become a major type of delivery form in the administration of biologics, specifically in case of therapeutic monoclonal antibodies (mAbs). From a regulatory point of view these devices are combination products and must be developed as medical products according to an ISO 13485 process, commonly as specified in the European Medical Device Regulation or in the 21 Code of Federal Regulations part 820.

As the medicine is administered by the patient themselves, patient compliance and thus human factors engineering plays a major role in the development of these products and is subject of extensive research [1–6]. However, not only human factors engineering, and the efficacy of the drug product is important for the well-being of the patient, the reliability of the device is also a major part. Reliability of the device is a crucial point for emergency devices as failure may result in patient death. But of course, it is also important in less critical applications to ensure correct therapy and patient well-being.

Most autoinjectors in use today contain a tense power spring to operate the injection [7] in connection with a pre-filled syringe (PFS) with a staked-in needle. The release mechanism of that power spring must also hold back the force of the tense power spring for the whole shelf life of the stored therapeutic drug product (i.e., 2 years). This contradictory design can be error prone and lead to malfunctions in autoinjectors. Furthermore, in the moment the release mechanism is triggered the power spring hits on the back of the plunger rod/stopper creating pressure spikes in the device e.g., up to 20 bar. This was demonstrated by Fitzgibbon et al. [8] and an in-depth analysis of pressure and stress transients can be found in an inspirating work by Veilleux et al. [9] from 2018. Veilleux et al. differed two phases after the activation of an autoinjector with PFS: the extrusion phase and the transient phase, while the extrusion phase is quasi-static regarding pressure and stress, the transient phase is characterized by three main events causing stress: the acceleration and deceleration of a syringe during automated needle insertion and the “impulsive application of a force on the plunger-stopper”. While the first and the second can be bypassed by autoinjector concepts that feature manual needle insertion, the latter is hardly avoidable for spring-based concepts.

To avoid this problem, we have developed a cartridge-based autoinjector concept where the force is retained on the primary packaging. The power spring directly presses on the plunger stopper of the cartridge which contains the drug product, and the force is withheld by the primary packaging material (i.e., the glass cartridge and the sealed rubber septum) thus composing a pre-pressurized device. Important to note is the double-ended needle system, which is part of the fluid pathway, but separated from the drug product in the cartridge during storage. The activation of this autoinjector is carried out by pushing the autoinjector on the skin to allow the piercing of the skin with the front end of the double-ended needle. This action subsequently initiates the piercing of the septum of the cartridge with the back end of the double-ended needle connecting the fluid pathway. This means that the device is by default in extrusion phase and bypasses the transient phase, as all three characteristics described by Veilleux et al. do not occur. The transient stress spikes on the primary packaging are avoided and replaced by a steady and lower stress for the whole duration of the shelf-life. The device and its toolset are described in detail in non-peer-reviewed magazine ONdrugDelivery [10, 11]. The goal of this publication is to show that general parameters for such a concept can be met by providing experimental data, specifically conducted to analyze weak points in standardized cartridges as primary packaging under continuous pressure (e.g., rubber septum stability) and demonstrate general feasibility of the primary packaging material for the application in the device (e.g. break-loose forces in acceptable ranges). It is especially important to test these parameters for standardized primary packaging as the vast majority of production chains only work with this material. Non-standardized material which corresponds to all requirements of the device concept may be easy to develop but could not be used without large investments in production stage and would therefore not lead to a benefit for the industry and successively the patient.

ISO cartridges for injectables (ISO 13926-1) are commonly made from Type I Pharma grade borosilicate glass defined in USP 660 and EP 3.2.1. Type I glass containers for injection have been subject to testing on mechanical and chemical properties [12]; however, most tests in recent years focused on glass delamination [13–15], which depends on chemical glass composition, hydrolytic resistance, glass processing history or the properties of the drug product [16]. The glass delamination process is increased with elevated temperatures and solvents / formulations with pH above 8.0 according to USP 1660. For a pressurized cartridge with an applied force at the plunger different types of experiments must be conducted to prove their suitability than what can be found in literature.

Regarding the drug product under pressure, there is literature dealing with cold pressure protein denaturation. At room temperature protein denaturation is documented at pressures around 5000 atm (~ 507 MPa) [17, 18]; however, it may already start much lower at around 100 MPa [19, 20]. The fact that mesophilic organisms only stop growing at around 40 to 50 MPa [20] indicates that proteins below that value should be largely immune to pressure. However, little is known about the behavior of IgG antibodies in physiologic conditions when a certain pressure is applied over a duration of days or more.

## Materials and methods

### Materials

#### Material numbering

In this publication some materials analyzed and compared are partly blinded. Companies are always denoted with a letter (e.g., Company A), while materials by that company are denoted with the company letter followed by a consecutive number. (e.g., Cartridge A1, Septum D1 and Plunger D2) The number is sequential to any material from the company, not to a specific type of material. (i.e., if there is a Septum D1, there may not be a Plunger D1, because #1 is already reserved for a Septum of Company D). As abbreviations we write CA1 for Cartridge A1, SD1 for Septum D1 and PD3 for Plunger D3. Materials and Cartridges for 1.5 mL ISO cartridges are marked with an asterisk (*), materials for 3 mL ISO cartridges have no additional marking. Septa are the same for 1.5 or 3 mL cartridges, therefore there are no septa marked with an asterisk. All used materials can be viewed in Table 1.

**Table 1:** List of materials

Company	Manufacturer	Cartridges	Septa	Plungers
A	glass	CA1, CA2*	n/a	n/a
B	glass	CB1, CB2*	n/a	n/a
C	glass	CC1	n/a	n/a
D	rubber	n/a	SD1, SD2, SD5	PD3, PD4*
E	rubber	n/a	SE1, SE2	PE3

#### Cartridge holders

**Fig. 1:**
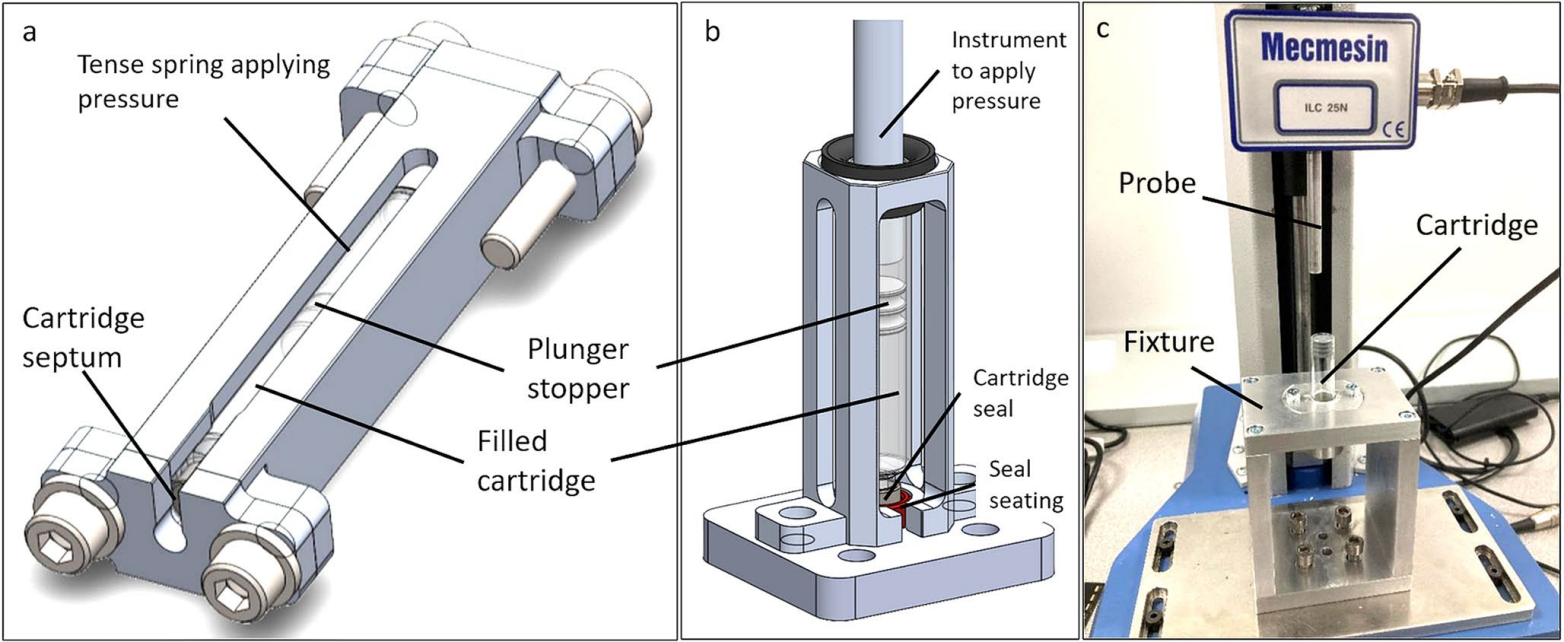
(**a**) Cartridge holder developed to keep cartridges under pressure for experiments. The device consists of the metal body, two end plates and screws on the outside. On the inside it has a seating for the cartridge, a filled cartridge, and the spring with spacers. (**b**) The image shows the cell into which cartridges were mounted to test for failure modes other than the septum. The cartridge is seated on the septum and enclosed in the mount. Pressure is applied from the back by a plunger rod with the Mecmesin instrument. (**c**) Mecmesin force testing system with cartridge in test fixture for measurement of glide and break-loose forces

For conducting the experiments under pressure custom fixtures to hold the cartridges under pressure were necessary. A fixture was designed, called ‘cartridge holder’ (CH), which can hold a single cartridge (see Fig. 1 (a)). The CH consists essentially of a lengthy body of metal with a tube-shaped hole in the size of a cartridge and two metal end plates. On the front side of the body a seating for the cartridge crimp cap could be inserted (different types of seatings are available). At the rear side of the body first the cartridge and then a spring can be inserted into the hole. Once the hole is filled the two metal end plates can be fixed to the body with screws and by fully tightening them the cartridge inside is put under pressure. A slit in the front-end plate allows the visual inspection of septum of the cartridge.

### Methods

### Statistical analysis

Mean values (MV) were calculated by arithmetic mean, standard deviations (SD) were calculated by unbiased sample variance.

Statistical t-test analysis was conducted with custom made python script previously used in [21]. It was verified against the open-source library Scipy Version 1.1 (Website: [22]) (Publication associated to Scipy version 1.0 [23]). All t-tests conducted in this publication are two-sided, two-sample Welch-tests with 95% confidence if not stated otherwise. An adjustment to the script was made for this publication to conduct a Shapiro-Wilk test prior to t-tests where sample sizes were large enough (*n* ≥ 10). Sample distributions with *p* ≤ 0.01 were considered to be significantly deviating from the normal distribution of the parent population.

### Experimental setups

#### Break-loose and glide forces

For measuring glide and break-loose force a Mecmesin Multi-Test 2.5 tensiometer with a load cell 879-002 (Mecmesin GmbH, Villingen-Schwenningen, Germany) was used. The cartridge was held by a custom-made test fixture. (see Fig. 1 (c))

To prepare an experiment the cartridge seal was removed, a plunger was inserted into the cartridge with by the use of a positioning block to put it into an exact location. The cartridge was then filled with a liquid and subsequently emptied before measurement. After placing the cartridge downwards in the fixture, the measurement could be started.

In the test program the probe-piston of the device was lowered until it was just above the plunger, then the probe was lowered at 25 mm/min until the load cell’s minimum force was measured. After that the device paused for 3 s with no further displacement. The probe was then raised − 0.2 mm with a rate of 25 mm/min and again a break of 3 s. The probe was then lowered again with a velocity of 160 mm/min until a force of 6 N was detected indicating the end of the cartridge.

**Fig. 2:**
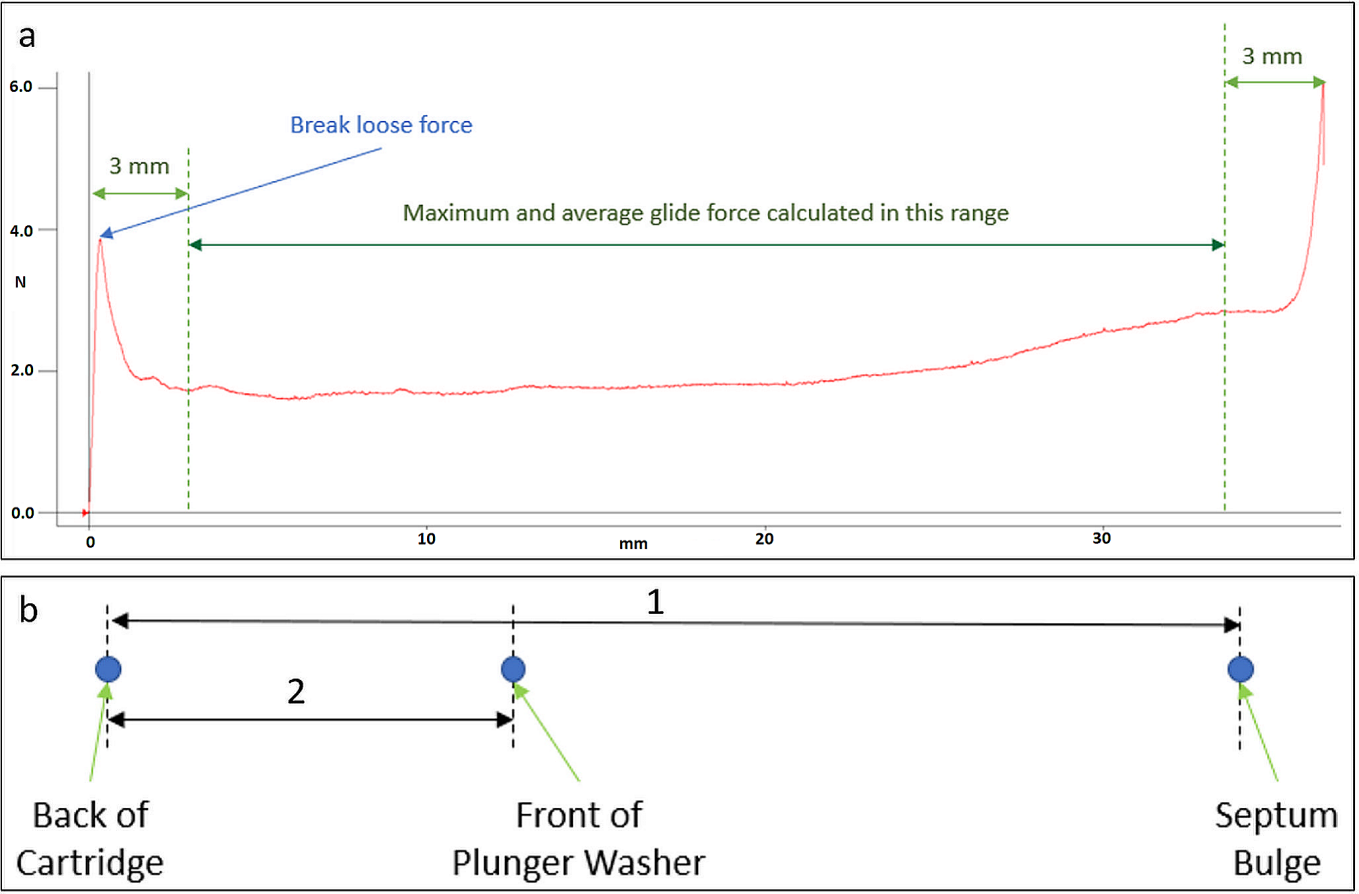
(**a**) Example graph of the measurement of break-loose and glide force of a plunger in a cartridge. The highest value in the initial peak is defined as the break-loose force while the averaged value of the marked area in the middle is defined as the glide force. (**b**) This figure shows the different measurements that were used to calculate key parameters of the septum and plunger changes under pressure. (1) is the distance from the rear end of the cartridge to the top end of septum bulge and (2) is the distance from the rear end of the cartridge to the top end of the plunger washer

Fig. 2(a) shows a graph resulting from the measurement described. The maximum force of the initial peak was defined as the break-loose force of the plunger in the cartridge, while the average value of marked region was defined as the glide force.

#### Septum pierce force under pressure

To test the pierce force of the septa, cartridges were locked in CHs pointing upwards. A needle was attached to the Mecmesin Multi-Test 2.5 tensiometer by the use of a 3D-printed adapter. The CH was placed below the tensiometer, facing upwards with the septum directly below the needle. The needle was lowered until directly above the cartridge septum. The program of the tensiometer lowered the needle 12 mm at a predefined velocity. A video camera was recording the plunger position. The force measurement of the tensiometer when the plunger was recorded to start moving was used as pierce force. For every experiment only a previously unpressurized cartridge and a new needle were used. Each septum was checked for leaking after its test to determine if there was a septum failure because of piercing under pressure.

#### Septum stability

Stability of the septum was seen as the most critical part in the device that has been constructed. Not only because it is a rubber part that is in the center of pressure but also because it must last this way for at least 2 years under real conditions of a medicine on the market. To assess the problem more than 1700 single measurements were conducted in 3 separate projects. Variable parameters were septum manufacturers as well as septum types (i.e., monolayer or bilayer). The seating surfaces of the septa and the pressure used was varied. Figure 2 (b) shows the basic parameters that were read from the experimental setup.

Cartridges were filled with liquid and placed in CH under fixed pressures. During the course of an experiment the bulging of the septum, the plunger position in the cartridge and the force acting on the cartridge were measured. Experiments were conducted for 42 days with time points on day 0 (before pressuring), day 0 after pressuring and days 1, 2, 7, 15, 32 and 42.

For long-term septum stability a small set of six cartridges was set up for a long-term stability test in CHs. It is a preliminary experiment to check general feasibility. Cartridge B2* with Septum E2 and a force of 50 N (1420 kPa) is used.

Further long-term tests as necessary for shelf-life of pharmaceuticals are not covered by this publication, however, an accelerated study according to ICH guideline Q1A(R2) [24] was conducted. For this study the septum bugle was observed at accelerated conditions for freezer, refrigerator, and room temperature conditions. The guideline does not define an accelerated setup for freezer condition but requests to test a single batch at elevated temperature for the time a drug product is used outside its proposed labeled storage condition. We used this condition as setup for our accelerated freezer experiments. With respect to this we tested:

1. 5 °C ± 3 °C (accelerated for the freezer).

2. 25 °C ± 2 °C at 60% ±5% relative humidity (accelerated for the refrigerator).

3. 40 °C ± 2 °C at 75% ±5% relative humidity (accelerated for room temperature).

The guideline requires a testing for at least 6 months for the second and third condition, the first condition has less strict rules, however, we tested it along with the other conditions. Cartridge A1 with septum E1 and plunger D3 were stored under the given conditions and at different pressurization levels (no pressurization, 960 kPa,1370 kPa) respectively. For condition 1 and 2, 6 cartridges per condition and pressurization level were tested at each time point. For condition 3, 8 cartridges were used per pressurization level and were tested at each time point. At the last data point (12 months) 9 cartridges for condition 3 were measured. The experiment was conducted for 12 months with the cartridges measured after 1, 3, 6, 9 and 12 months. This specifically allowed to monitor the changes of the septum bulge after the initial growth phase of the first 14 days.

#### Secondary pressure failure mode

To detect secondary pressure failure mode after the septum, the cartridge was placed in a specially enclosed mount that fully supported the septum on the bottom, and the plunger was compressed with 2.5 kN load cell to increase the pressure (see Fig. 1 (b)). Before starting the experiment, cartridges were filled with methylene blue solution (1.9 mL for 3 mL cartridges and 1.1 mL for 1.5 mL cartridges).

To run a test a loaded mount was attached to the tensiometer (Mecmesin Multi-Test 2.5). The force was brought to 100 N by lowering the probe with 3 mm/min. Then force was increased stepwise with 20 N per step with the same velocity. After each increment a 6 s break followed in which crimp cap and plunger region were inspected for leaking and the glass cartridge was inspected for fracture. End point of an experiment was 20 mm of movement, a maximum force of 2000 N or any failure of the cartridge.

#### Drug product stability

Damage to a drug product due to a continuous pressure of up to ~ 1400 kPa as it is the case in the device setup for which the experiments were conducted is unlikely as described in the introduction. Nonetheless, a general test with a commercially available mAb drug product was considered to be beneficial for characterizing the device properties. In this context not only long-term pressure influence but also the influence of the extrusion through the fluid pathway of the device was tested.

For these experiments a commercially available Adalimumab drug product (Yuflyma®, Celltrion Inc [25]) with a concentration of 100 mg Adalimumab / 1 ml was obtained and tested with a release testing protocol in a GMP environment. In total 100 1.5 mL cartridges were filled with 0.8 mL Adalimumab drug product. A total of 48 of these test samples were used evaluating the fluid pathway. Another 48 samples were split into 4*12 batches of which 3 were kept under a pressure of nominally 1357 kPa. For each time point (3, 6 and 12 months) 12 cartridges were tested. The 12 unpressurized cartridges were opened by removing the cap and tested in order to verify that the filling method did not produce an artifact. The remaining 4 cartridges were kept as backup. All samples were stored in a climate chamber according to ICH Q1A(R2) at 5 °C ± 3 °C [24].

Evaluation of the fluid pathway was conducted with two different initial pressures (1357 kPa and 557 kPa) with two needle variants different in their diameters (27 G and 29 G). For simulation of the fluid pathway components of the corresponding autoinjector devices were used. The 48 samples were distributed to 4*12 samples.

The commercial Adalimumab drug product contains: Adalimumab mAb, Acetic acid, Sodium acetate trihydrate, Glycin, Polysorbate 80 and Water for injection.

To analyze the mAb drug product a set of experiments was conducted:

- Size Exclusion Ultra Performance Liquid Chromatography (SEC).

- Capillary Electrophoresis (CE).

o Electrophoretic Purity (Reducing (R)) analysis procedure (with sodium dodecyl sulphate).

o Electrophoretic Purity (Non-reducing (NR)) analysis procedure.

- Subvisible Particulate according to USP 787.

These analytic methods were chosen because they are being used for the commercial release of protein drug products upon production. Size exclusion chromatography is able to demonstrate if protein agglomerates have formed. The percentage of the antibody monomer is supposed to be above 98% and larger structures below 2%. In the reducing electrophoresis the mAbs are denatured, meaning that disulfate bridges are destroyed and as a result single polypeptide chains are visible in the gel. It is checked for the combined amount of antibody high and low molecular weight chains which may not be less than 95% to ensure no other proteins are present in the drug product. The non-reducing electrophoresis shows if there are denatured antibody chains present. Lastly the sub-visible particle analysis is conducted for characterizing large visible contaminants. Here less than 6000 particles with a size of ≥ 10 μm per container and 600 particles with a size of ≥ 25 μm per container are permitted.

## Results

### Break-loose and glide forces

#### Unpressurized (first batch)

To understand the forces necessary to move plungers, a set of 26 force measurements were conducted. All except for 3 experiments were conducted with cartridges wetted with NaCl solution, the 3 remaining experiments were done with glycerol solution. 6 cartridges were tested with a velocity of 50 mm/min, all other tests were tested with 160 mm/min. In all cases Company D Plunger PD3 was used with 3 mL cartridges and Plunger PD4 with 1.5 mL cartridges. The only difference between the plungers were their outer diameter to fit into the cartridges. All experiments are listed in Table 2.

**Table 2:** Glide force and break-loose force experiments with Company D Plunger PD3 for 3 mL cartridges and Plunger PD4 for 1.5 mL cartridges

#	Cartridge	Velocity [mm/min]	Liquid	*n*	MV GF [*N*]	SD GF [*N*]	MV BLF [*N*]	SD BLF [*N*]
1	CB1	160	NaCl 0.9%	6	2.2	0.43	3.73	0.31
2	CB2*	50	NaCl 0.9%	6	3.8	0.80	4.45	0.38
3	CB2*	160	Glycerol	3	4.6	0.40	4.35	0.15
4	CB2*	160	NaCl 0.9%	11	5.1	0.67	5.42	0.51

#### Pressurized (second batch)

A set of tests was conducted with cartridges stored for 1, 3 and 6 months under different forces ((0 N control), 70 N and 100 N) prior to testing. For the display and analysis of the results different storage conditions were pooled according to pressure on the cartridges, as the underlying question of the experiment is whether glide force changes due to storage under pressure. A separate set of 60 experiments was conducted with 3 months under pressure. Here 30 tests cartridges of Company A (3 mL) and 30 cartridges (3 mL) of Company C were compared. Averages and standard deviations can be seen in Table 3.

**Table 3:** Glide forces with Plunger PD3, Cartridge CA1 and a velocity of 160 mm/min after being stored under pressure for 1, 3 or 6 months

#	Cartridge	pressurized [months]	Liquid	*n*	pressure [kPa]	MV GF [*N*]	SD GF [*N*]
5	CA1	3	NaCl 0.9%	30	1370	2.44	0.28
6	CC1	3	NaCl 0.9%	30	1370	1.83	0.53
7	CA1	1	NaCl 0.9%	10	0	2.35	0.13
8	CA1	1	glycerol	9	959	1.99	0.40
9	CA1	1	NaCl 0.9%	10	1370	2.65	0.38
10	CA1	3	NaCl 0.9%	10	0	1.96	0.45
11	CA1	3	glycerol	9	959	1.89	0.19
12	CA1	3	NaCl 0.9%	10	1370	2.56	0.47
13	CA1	6	NaCl 0.9%	10	0	2.04	0.34
14	CA1	6	glycerol	9	959	2.20	0.34
15	CA1	6	NaCl 0.9%	10	1370	2.80	0.53

For experiments #5 and #6 also break-loose forces were measured. In #5 the mean break-loose force was 5.54 N with a SD of 0.35 N. In experiment #6 the mean value was 5.55 N with a SD of 0.44 N.

### Septum pierce force under pressure

Pierce force under pressure and leakage/rupture upon piercing under pressure were analyzed. In total 444 septum tests were conducted. Among those were 304 where the test was conducted on the same day the cartridge was pressurized, and 140 experiments where the cartridges were pressurized for 42 days before the test. In no case septum rupture or leakage occurred upon piercing. Four different septa from 2 different manufacturers were tested with 5 different needles from either Gerresheimer (Düsseldorf, Germany) or Europin (Trenčín, Slovakia) with different gauge diameters and cuts. The cartridges were either pressurized with 1370 kPa or with 410 kPa (nominal pressure) except for 10 unpressurized controls. Piercing speed was either 20–200 mm/min except for 5 cartridges which were tested with 1000 mm/min. Mean Pierce forces of the respective experiments were between 4.04 N and 13.41 N.

A conclusive subset is presented in Table 4. All experiments in the table were conducted with a Gerresheimer 22 Gauge 510.12341 needle (Gerresheimer AG, Düsseldorf, Germany) at a pierce speed of 200 mm/min.

**Table 4:** Subset of different monolayer septa pierced with a Gerresheimer 22G needle at 20 mm/min, a non-seating surface of 3.14 mm² and a (measured) pressure of 1340 ± 10 kPa

#	Septum	pressurized storage [days]	sample size	MV [*N*]	SD [*N*]	Max [*N*]	Min [*N*]
1	SD1	0	9	11.01	2.12	13.32	5.69
2	SE1	0	12	6.50	0.46	7.47	5.79
3	SD2	42	20	8.75	0.78	10.02	6.92
4	SD1	42	12	13.13	2.15	17.02	10.45
5	SE1	42	19	6.62	0.97	8.00	4.06

### Septum stability

In order to prove septum stability under pressure a large test program was conducted. In the beginning bilayer septa were tested which did not withstand the pressure. Septum D5, a bilayer septum, for example withstood a pressure of 1370 kPa for only 15 min before rupturing. In a test with a pressure of 1030 kPa the septum withstood about 1 h. Monolayer septa on the other hand bulged but were stable. For this reason, all the following results described have been obtained with monolayer septa. They were used with a non-seating surface of 3.14 mm². Septum bulging over time resulted in a growth curve of similar shape for all experiments except for experiment #2. For most experiments not only septum bulge, but also plunger position and applied force was measured.

**Fig. 3:**
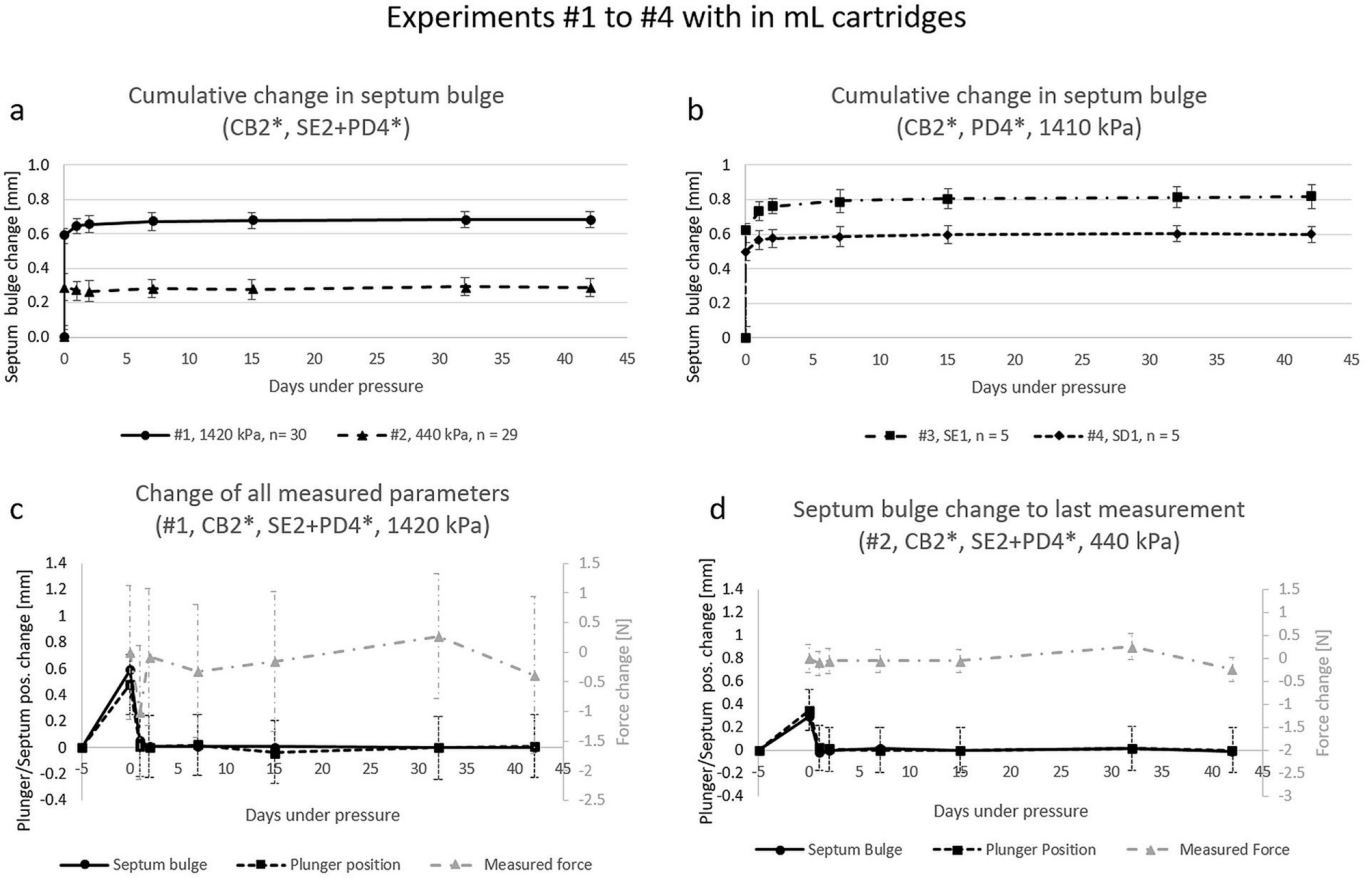
Graphical depiction of the results of experiments with 1.5 mL cartridges. (**a**) shows the change in septum bulge over time of experiments #1 and #2. (**b**) shows the septum bulge of two smaller experiments #3 and #4. (**c**) shows the changes at each time point in relation to the last measured time point for septum bulge, plunger movement and measured force of experiment #1, (**d**) is the same as c) for experiment #2. The values of ‘Day − 5’ are the unpressurized values, they were also measured on day 0 and are displayed as day − 5 for an improved readability of the diagrams only

Four experiments with 1.5 mL cartridges were conducted with a fill volume of 1 mL. Two smaller experiments (#3 and #4) with *n* = 5 that can be seen in Fig. 3 graph b) and 2 main experiments with *n* = 30 (#1) and *n* = 29 (#2), which can be seen in Fig. 3 graph a), c) and d). a) shows the septum bulge of the two main experiments (#1, #2) over time. The main difference between the two experiments is the applied pressure. #1 is conducted with 1420 kPa pressure and has an average septum bulge of + 0.68 mm after 42 days and #2 with a pressure of 440 kPa and an average septum bulge of + 0.29 mm after 42 days. Standard deviations in all 4 experiments are below 0.1 mm for all values. The experiments #3 and #4 were conducted at the same pressure as #1 but with other septa to get a basic overview if other septa behave the same way. Both septa of #3 and #4 showed the same bulging curve with different extent (+ 0.81 mm and + 0.60 mm after 42 days). Figure 3 graph c) and d) show additional information for experiments #1 and #2 respectively. Here the relative change of the septum bulge, plunger position and force in comparison to the measurement before is shown. This means the first data point for septum and plunger is the difference between the non-pressurized measurement and the first measurement after pressurization. The force curve starts one measurement later as considering the non-pressurized measurement would not make sense. Therefore, septum and plunger curve start at 0 while the force curve starts with a value greater than 0. The graphs of c) and d) allow to see the changes in septum bulge and plunger position in relation to changes in pressure in the cartridge. The septum bulge curves of c) and d) do not show standard deviation as it is shown in a) already and this way enhances readability of the graph.

**Fig. 4:**
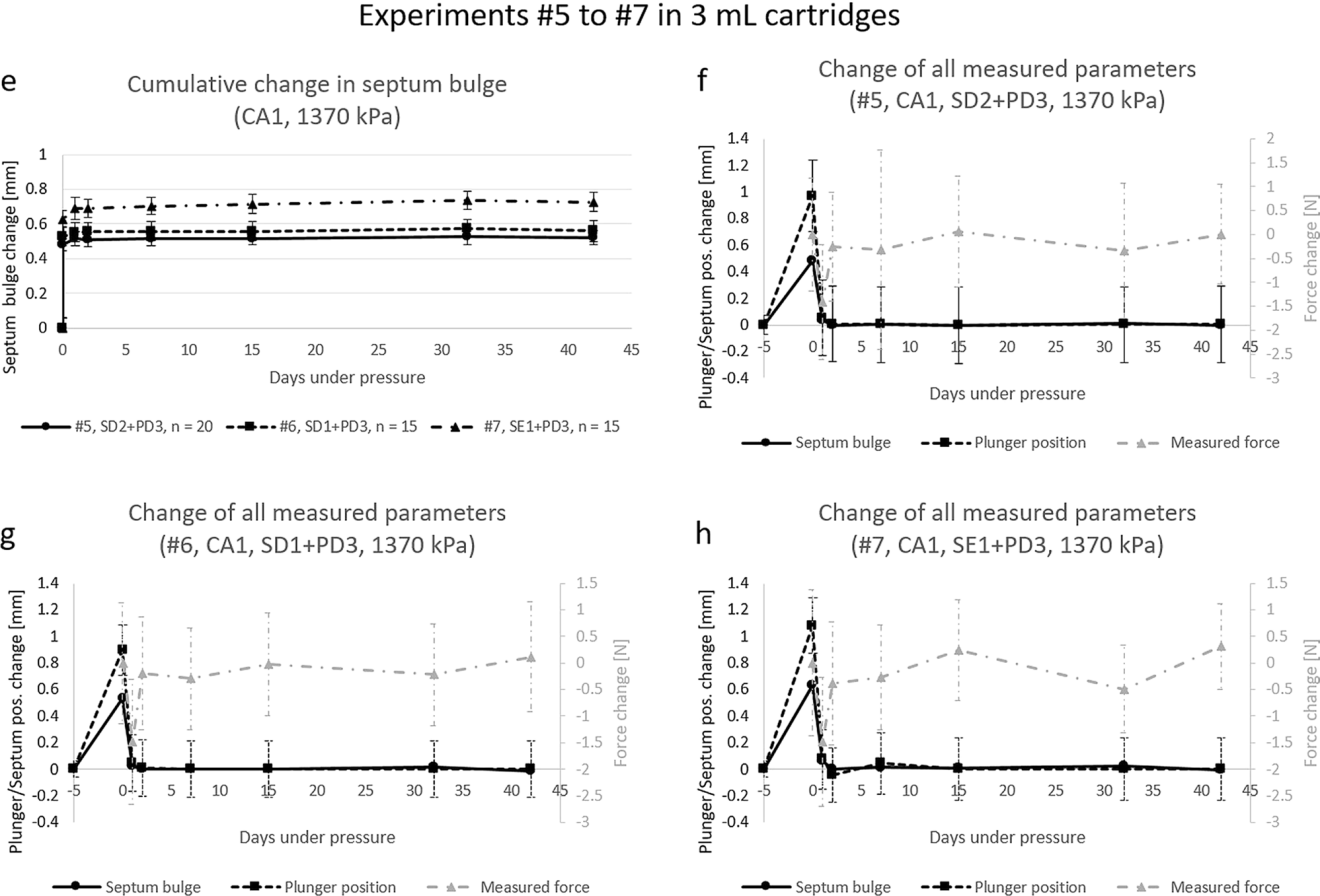
Graphical depiction of the results of experiments with 3 mL cartridges. (**e**) shows the change in septum bulge over time of experiments #5, #6 and #7. (**f**) shows the changes at each time point in relation to the last measured time point for septum bulge, plunger movement and measured force of experiment #1, (**g**) and (**h**) are the same as f) for experiments #6 and #7 respectively. The values of ‘Day − 5’ are the unpressurized values, they were also measured on day 0 and are displayed as day − 5 for an improved readability of the diagrams only

Three experiments with 3 mL cartridges and 2 mL fill volume were conducted with *n* = 20 for #5 and *n* = 15 for #6 and #7 with the goal to see differences between different septa. Figure 4 graph e) shows the septum bulges growth over time with the same shape as in experiments #1, #3 and #4. All experiments were conducted at 1370 kPa pressure and thus are quite similar to the experiments with 1410 and 1420 kPa. Septum bulges extended to + 0.52 mm for #5, + 0.56 mm for #6 and + 0.73 mm for #7. Standard deviation for septa were for all data points at all time points below 0.1 mm. The graphs f), g) and h) allow to see changes in the septum bulge and plunger position in relation to changes in the force constituting the pressure in the cartridges. The graphs have been prepared analogously to graph c) and d) in Fig. 3, which were described in detail before.

**Table 5:** Absolute septum bulge increase and relative change between the last two data points

Experiment	Cartridge	Pressure [kPa]	Septum	Plunger	*n*	Septum position Day 42 [mm]	Change Day 32 to Day 42 [mm]
#1	CB2*	1420	SE2	PD4*	30	+ 0.68	+ 0.00
#2	CB2*	440	SE2	PD4*	29	+ 0.29	− 0.01
#3	CB2*	1410	SE1	PD4*	5	+ 0.81	+ 0.01
#4	CB2*	1410	SD1	PD4*	5	+ 0.60	+ 0.01
#5	CA1	1370	SD2	PD3	20	+ 0.52	− 0.00
#6	CA1	1370	SD1	PD3	15	+ 0.56	− 0.01
#7	CA1	1370	SE2	PD3	15	+ 0.73	− 0.01

Table 5 shows an overview on the different experimental setups presented in this section. For each setup the used primary packaging parts, pressure and number of cartridges can be seen. Furthermore, the total septum bulge increase after 42 days and the difference in increase/decrease between the Day 32 and Day 42 time points is shown.

#### Long-term septum stability

**Fig. 5:**
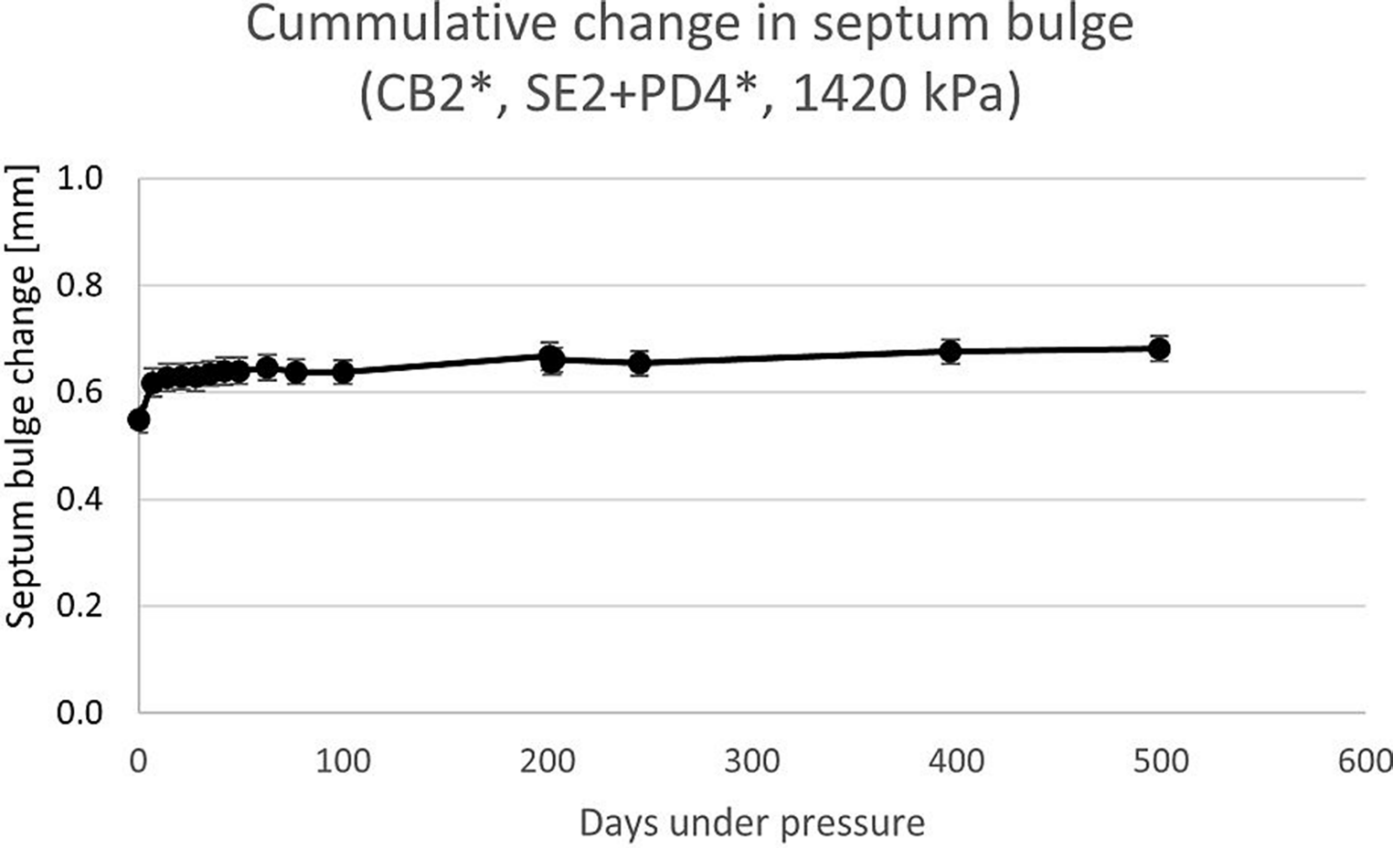
Change in septum bulge over 499 days under pressure

Figure 5 shows the result of the long-term septum stability test. The measurement of Day 499 shows an average septum bulge of 0.68 mm at a standard deviation of 0.02 mm. The change from data point of day 397 (which is one before day 499) is 0.006 mm and thus within standard deviation.

**Fig. 6:**
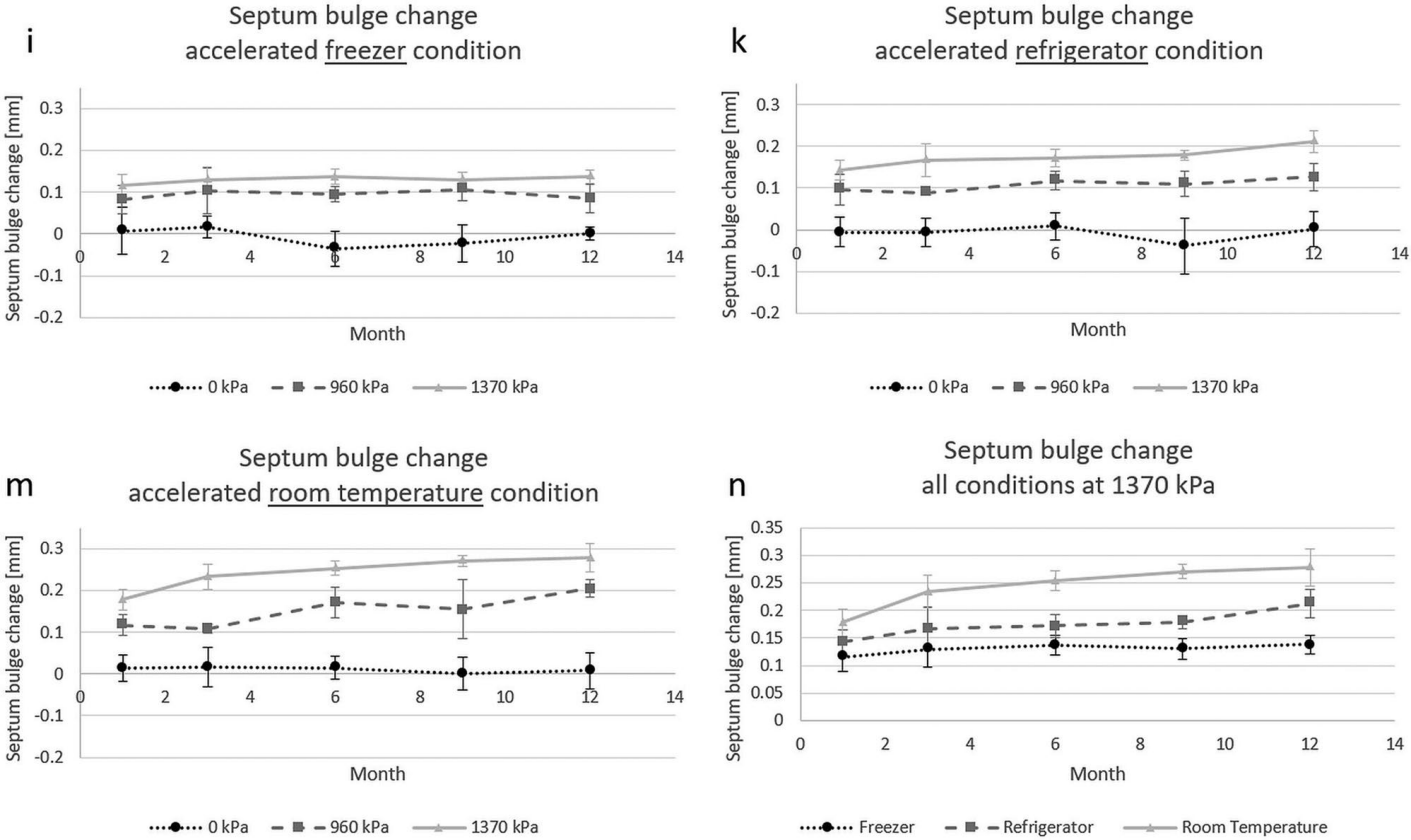
Graphs (**i**), (**k**) and (**m**) show the change of the septum bulge in the course of the accelerated aging experiment according to storage conditions defined in ICH guideline Q1A (R2). The different lines represent different levels of pressure in the cartridges over the whole duration of the experiment with the black dotted line being the control with no pressurization. Graph (**n**) is a summary of the three different storage conditions at the highest level of pressure. Letter ‘j’ and ‘l’ were not used to avoid confusion with ‘i’

The changes of the septum bulge in the accelerated testing is shown in graphs i), k) m) and n) in Fig. 6. Absolute maximum of changes was an increase to + 0.35 mm for one cartridge of the 1370 kPa accelerated room temperature batch at 12 months pressurization.

In order to see the changes between the different curves a linear regression function (y = ax + b) was calculated for each curve. The coefficients a and b of the graphs for the different conditions are shown in Table 6. ‘ACC FRZ’ is the accelerated freezer condition which also equals a non-accelerated refrigerator condition. The ‘ACC FRG’ is the condition for accelerated refrigerator which equals the non-accelerated room temperature condition. Lastly the ‘ACC RT’ is the accelerated room temperature condition. The slope of the linear regressions (coefficient a) can be used as a measure of change for the different conditions independently from the change during the initial bulging phase. The regression lines further allow to predict the y-value for 24 months (Y24) for room temperature and refrigerator conditions according to ICH guidelines Q1A (R2) and Q1E [26].

**Table 6:** Linear regression coefficients of graphs in i), k) and m) shown in Figs. 6 and 24 months extrapolation

Pressure [kPa]	ACC FRZ			ACC FRG			ACC RT	
	a	b	Y24 [mm]	a	b	Y24 [mm]	a	b
0	-0.0017	0.0041	N/A	-0.0006	0.0041	N/A	-0.0001	0.0172
960	0.0002	0.0931	0.0979	0.0029	0.0898	0.1594	0.0080	0.1021
1370	0.0015	0.1206	0.1566	0.0053	0.1415	0.2687	0.0082	0.1921

### Secondary pressure failure mode

During the experiments for secondary pressure failure mode some cartridges leaked small amounts of liquid from the crimp cap and in some cases, liquid leaked behind the first rib of the plunger stopper. For the leaking crimp caps, it happened only with CA2* cartridges of one specific lot. We therefore assumed that this study artifact was caused by a quality issue in the manufacturing process. The plunger stopper was introduced into the cartridge from behind with a tube or a cannula to allow pressure compensation when pushing the plunger stopper inwards. The leaking behind the first rib of the plunger stopper could be attributed to a certain filling method. Once the method was adjusted no more leaking could be determined and as preparation artifact it is not of importance for the purpose of the experiments. Once the method was adjusted no more leakage could be observed. In most cases, when leakage at the crimp cap or the plunger stopper occurred, the experiments could be continued until a total failure of one component of the primary container could be reached. Crimp caps of the cartridges deformed at pressures between 5500 and 6800 kPa but did not break. The total failure was in all cases the glass part; Table 7 shows the mean pressures at which cartridges break along with their standard deviation and the minimum value at which a cartridge in the test set broke. The lowest value in all experiments at which a cartridge broke was 4922 kPa.

**Table 7:** Results of secondary failure mode experiments

	CB1 + PD3	CB1 + PE3	CA2*+PD4*	CA1 + PE3
MV [kPa]	11,375	11,139	11,479	7899
SD [kPa]	2743	3896	1116	830
MIN [kPa]	7656	6836	9226	4922

### Drug product stability

**Table 8:** Results of the analysis of the Adalimumab drug product with size exclusion chromatography, capillary electrophoresis and subvisible particle analysis

month		SEC			CE *R*		CE NR		SP	
		HMW (%)	Monomer (%)	LMW (%)	LC + HC (%)	NGHC (%)	Pre-Peaks (%)	IgG (%)	Particle Size ≥ 10 μm	Particle Size ≥ 25 μm
0	control OC	0.65	99	0.34	99.4	0.65	2.6	97.4	615	15
	filling/shipping control	0.68	99	0.36	99.5	0.52	3	97	531	1
	21 *N* − 27G	0.69	98.9	0.4	99.3	0.67	2.6	97.4	159	1
	21 *N* − 29G	0.68	98.9	0.38	99.4	0.62	2.8	97.2	2427	279
	50 *N* − 27G	0.72	98.9	0.38	99.4	0.65	2.7	97.3	233	1
	50 *N* − 29G	0.7	98.9	0.37	99.3	0.66	2.6	97.4	99	0
3	control OC	0.72	98.9	0.41	99.3	0.68	2.3	97.7	432	8
	sample	0.73	98.8	0.43	99.3	0.69	2.6	97.4	191	8
6	control OC	0.74	98.9	0.4	99.3	0.71	2.6	97.4	641	1
	sample	0.78	98.8	0.42	99.3	0.68	2.8	97.2	388	9
12	control OC	0.81	98.8	0.43	99.3	0.71	3.9	96.2	527	17
	sample	0.88	98.7	0.42	99.3	0.73	3.7	96.3	19	1
Requirement		≤ 2.0	≥ 98.0	-	≥ 95.0	-	-	-	≤ 6000	≤ 600

The Adalimumab drug product was filled into 12 cartridges for each experiment and expelled through the fluid pathway constructed from device components. This served to see whether a degradation of the mAb occurs, specifically due to the needle or the pressure. It was conducted at two different force levels (21 N and 50 N) and with two different needles (27G and 29G).

The long-term stability of the Adalimumab drug product was tested at four different time points (month 0, 3, 6 and 12) (see Table 8). At each time point the drug product from the original container (OC) and one which was kept under pressure (sample) was tested (50 N/29G). For the 0 months time-point only an OC control was tested, the 50 N/29G sample of the degradation experiment served as month 0 ‘sample’ in comparisons with time points month 3, 6 and 12.

**Fig. 7:**
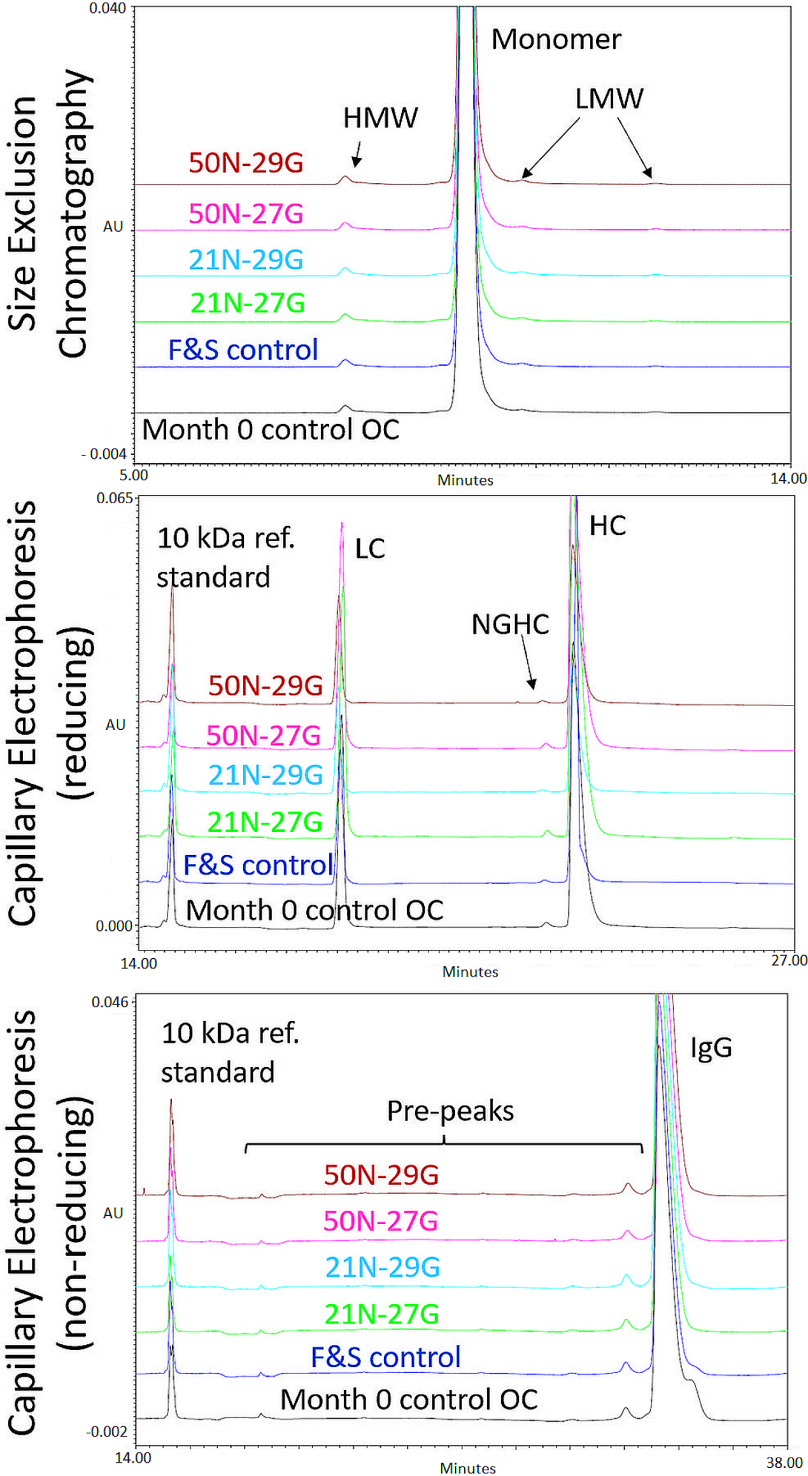
This figure shows the chromatograms and electrophoresis diagram of the analysis. For Size Exclusion Chromatography high and low molecular weight (HMW/LMW) and the monomer content are marked. For reducing capillary electrophoresis light chains (LC), heavy chains (HC) non glycosylated heavy chains (NGHC) are marked. For the non-reducing capillary electrophoresis pre-peaks and the IgG are marked. For both electrophoretic methods a 10 kDa reference standard was used. The drug product was expelled at 4 different configurations (regarding force in Newton (N) and needle gauge (G)); furthermore, the drug product from the original container (OC) was tested as control. The F&S control tested that filling and shipping did not have an influence on the drug product

**Fig. 8:**
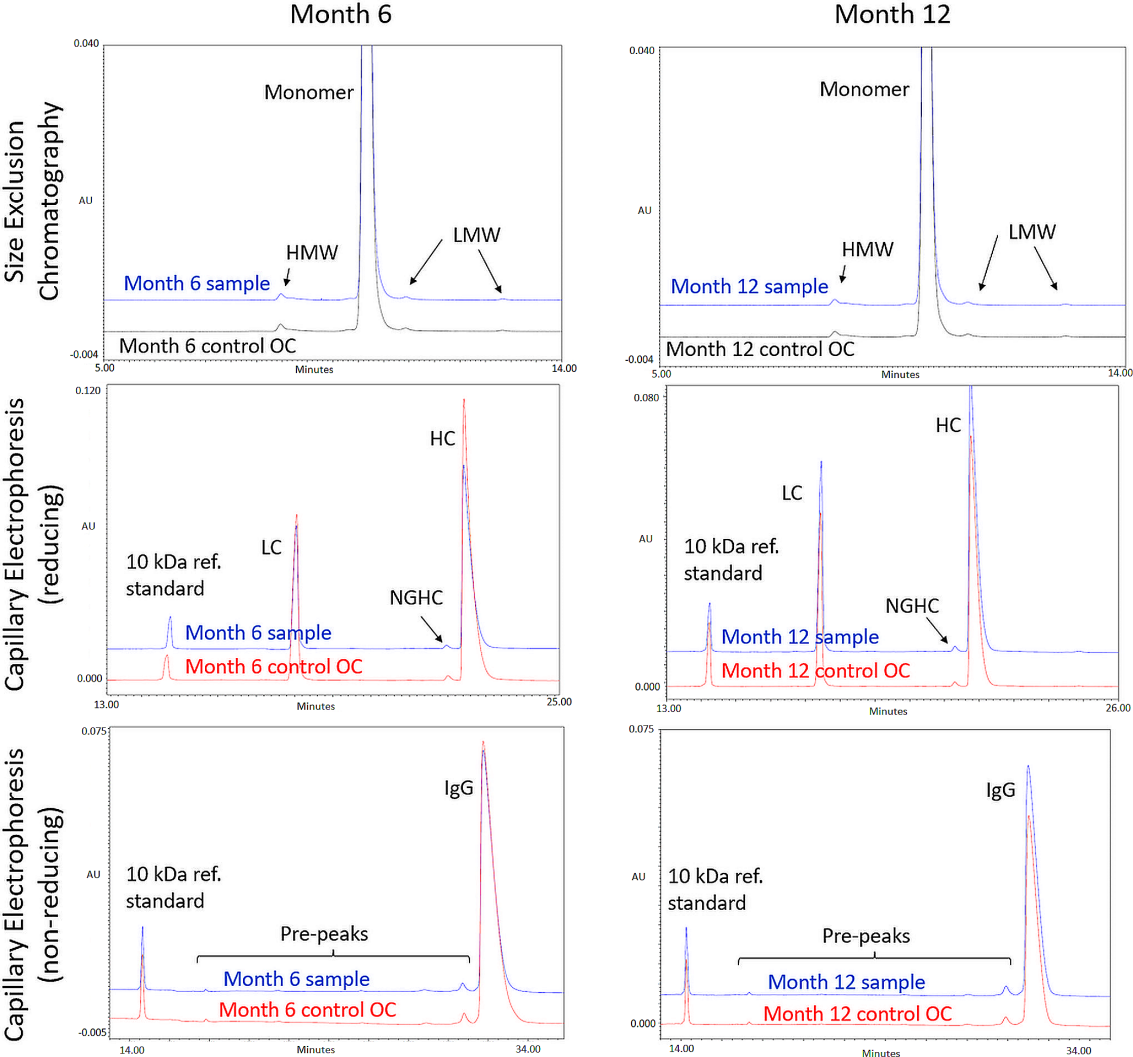
This figure shows the chromatograms and electrophoresis diagrams of the analysis for month 6 and 12. For Size Exclusion Chromatography high and low molecular weight (HMW/LMW) and the monomer content are marked. For reducing capillary electrophoresis light chains (LC), heavy chains (HC) non glycosylated heavy chains (NGHC) are marked. For the non-reducing capillary electrophoresis pre-peaks and the IgG are marked. For both electrophoretic methods a 10 kDa reference standard was used. A sample that was stored under pressure was expelled (50 N/29 needle gauge) and tested. As control the drug product from the original container (OC) was tested

The mAb drug product was filled into another 12 cartridges and retrieved by removing the cap (not by expelling through the fluid pathway). This was done to test if the filling method or the cartridge shipping had any impact on the drug product. Four methods were used for analysis: With size exclusion chromatography the distribution between mAb monomer, high-molecular weight (HMW) residues and low molecular weight (LMW) residues was determined. With reducing capillary electrophoresis, the cumulative light chain and heavy chain content (LC + HC) and content of non-glycosylated heavy chain (NGHC) was determined. The non-reducing capillary electrophoresis was used to determine pre-peaks and the IgG as whole. Figures 7 and 8 show the chromatograms and the diagrams of the electrophoresis. Diagrams for month 3 are not shown as they do not contain any additional information value. Subvisible particles was analyzed according to USP 787.

## Discussion

### Break loose force and glide force

Break-loose and glide forces were first analyzed to get an estimation for the necessary forces. Different setups were tested in smaller sample sizes to see which parameters change values on large scale. The small datasets only allow limited comparability. In this sense a t-test reveals a significant difference in break-loose force between the 3 mL (experiment #1) and the 1.5 mL (experiment #4) cartridges (*p* = 3.05*10^− 5^). This is not uninteresting as both cartridges are from the same company (Company B) and the two plungers are basically the same plungers just for different cartridge sizes.

The two large datasets of Experiments #5 and #6 would be interesting to compare as their BLF are very similar while the GF differ by almost 25%. Unfortunately, a Shapiro-Wilk test was significant for Experiment #5 such that a t-test cannot be conducted reliably. The BLF force of experiments #5 and #6 does not seem to be much different compared to the experiments #1 - #4. In literature Yoshino et al. [27] documented a change of BLF over storage time for systems containing liquid silicone oil. It seems that the baked-on silicone oil is more resistant to this change. Funke et al. [28] documented an increase for baked-on silicone oil for BLF but clearly showed that there is a difference regarding layer thickness. In the existing experiments a difference in BLF due to storage under pressure cannot be seen, but also was only tested for 2 weeks.

A further comparison between experiments reveals a significant difference (*p* = 4.96*10^− 6^) between glide forces of 3 mL and 1.5 mL cartridges. This is conclusive with a significant difference in the BLF between the two datasets, yet it was not tested consecutively. Similarly, there is a significant difference (*p* = 0.006) between using a test speed of 50 mm/min or 160 mm/min for measurement. Despite the significance in the two cases, the differences are so small that there is no influence for the purpose of this study. However, it should be kept in mind at other places in device development where it might make a difference.

Comparing experiments #7 and #15 we can have a look into a change due to storage under pressure. Indeed, glide forces of both experiments are significantly different (*p* = 0.012). To further specify the difference, experiments #7 and #13 were tested for a significant difference in a consecutive t-test (with alpha-error correction) to see whether the change is due to pressure. Unfortunately, the test was not significant such that further conclusion cannot be drawn here.

Significant differences between some of the datasets give an interesting insight into factors that contribute to changes. For the purposes of this project the maximum tolerable BLF was set to 15 N which is almost three-fold higher as the highest mean values measured. In case of GF the maximum tolerable GF was set to 10 N being 2-fold to 5-fold higher than mean GF values measured. We therefore conclude that impacts of different primary packaging on BLF and GF are too small to make an impact on the purpose of the project.

### Septum pierce force under pressure

The main concerns for the pressurized septum during piercing was that either the septum blasts due to a weakness in the material introduced by the needle cutting into it, or the drug product leaking from the cartridge outside the fluid pathway. In none of the 444 experiments that were conducted either effect was observed. Therefore, we consider the issue as unproblematic. A main issue here may be the non-seat engaging surface which was lowered to an area of 3.14 mm^2^ and the pressure on the cartridge which held it in its mount.

Table 4 shows an excerpt of the conducted experiments with datasets that are comparable with each other. Experiments #1 and #4 are both under pressure during piercing, they differ only by the 42 days of storage under pressure. Standard deviation in the pierce force is the same; however, the average is 2 N higher. Likewise maximum and minimum values are higher as well. A t-test revealed that there is a significant difference between both datasets with *p* = 0.041, so pressure clearly makes a difference regarding the pierce force for this type of septum.

When comparing Experiments #2 and #5 a real difference cannot be seen, even though mean and max values are higher in the 42 days experiment, the min value is higher in the 0 days experiment. The t-test we conducted here did not conclusively proof a difference between the two samples.

Also, an interesting point is to compare experiments #4 and #5. The data is very different for both sample sets. A t-test supports the impression, indeed both samples are significantly different with *p* = 2.667*10^− 10^. So septa from different companies make a very clear difference. When looking at experiments #3 and #4, which are two different septa from the same company after 42 days of pressure, the values also appear to be different, but a t-test did not deliver a conclusive result on a difference between the two sample sets.

### Septum stability

The septum bulge growth curves of all but one experiment are very similar to each other. The exception, experiment #2 (shown in Fig. 3), has a slight decrease of septum growth at day 1 (data point 3). Considering the change of pressure in graphs c), d), f), g) and h) of Figs. 3 and 4 there is always a drop in force on day 1. Interestingly for all the high-pressure experiments (1370–1420 kPa) this drop is larger than 1 N, for the low-pressure experiment #2 it was only recorded to be 0.1 N. The reason why the septum bulge reduced may be due to the low overall pressure in the experiment compared to the other experiments. However, the change in septum bulge on day 1 for #2 was on average − 0.024 mm while the standard deviation was 0.055 mm. The reason for this behavior remains unclear, but it is likely that the decrease is an error due to the optical measurement method.

The general shape of bulge growth has a large change in the beginning and is going into a steady state after about 14 days. If we look at the data points of 32 and 42 days we can see that the difference between those two in all experiments on average is ± 0.01 mm. (see Table 5) This clearly shows that the septa go into a stable state. The extent of the septum bulge is dependent on pressure that is used which can be seen when comparing experiment #2 and #7 (average septum bulge increase after 42 days of 0.29 mm vs. 0.73 mm). If we consider all experiments with pressures between 1370 and 1420 kPa to be comparable we can say that septa of company E on average bulge more (0.68–0.81 mm) than septa of company D (0.52–0.60 mm). When taking septum pierce force into account where company E septa had on average lower pierce forces than company D septa it could be assumed that company E generally uses a softer material.

The force and thus the pressure in the cartridge fluctuate in the first days after the pressurization by less than 2 N on average for all cases. After this initial fluctuation the force goes to a continuous state with ± 0.5 N difference in the first 42 days. The deviation here is likely due to the experimental setup and may originate from friction and slight changes of the angle of the cartridge in the CH. The large standard deviation may be due to the spring itself which comes with tolerance of ± 10 N (manufacturer’s information) from production. In reality, however, the measured spring force deviations are around ± 2 N.

Similar to the septa the plunger position stabilizes within the first 14 days, which can be seen in graph c) and d) of Fig. 3 and graph f), g) and h) of Fig. 4. The plunger movement of roughly 1 mm on the first day can probably be attributed to compression and dissolution of a small gas bubble which resulted from filling. Other factors may be the compression of the rubber parts and the displacement of the septum. Fluid compression (2 mL H_2_O, bulk modulus of 2.08 GPa, 1370 kPa pressure) may result in only 0.018 mm plunger movement and is thus not relevant.

All septa used in the presented experiments were monolayer septa. Bilayer septa were not used as in very early experiments it was shown that those septa cannot withstand the pressure. They break within a day of pressurization depending on the pressure applied. We presume that two thinner layers of rubber, as in case of the bilayer septa, is much less stable than one thicker layer of rubber, as in case of the monolayer septa. With respect to the purpose of the study this is not an issue for the device that is being developed. Monolayer septa are for single piercing while bilayer septa are made for multiple piercing into the primary container. The device that is developed is a single-use and single-dose device. Furthermore, reducing the non-seating surface of the cartridge septum to 3.14 mm² helped to further stabilize monolayer septa.

#### Long-term septum stability

The long-term septum stability experiment shows that it is in principle feasible to store the cartridges under 1420 kPa pressure for a longer time. A change in the septum bulge of the last two data points is within their standard deviations, while the curve follows a logarithmic pattern and still grows slightly over time we consider it to be stable for our purpose. The experiment will run until 2 years have passed which is the storage life for common medicines.

Regarding the accelerated experiment a comparison of the graphs i), k) and m) shows that there are no differences between the storage conditions regarding the shape of the curves. The graph for the 1370 kPa always has the highest septum bulge and the graph for 960 kPa is between the two other graphs. The only clearly visible difference is the extent to which the septa bulge. When comparing the slopes displayed in Table 6 it becomes visible that for the samples without pressurization the storage condition seems to have an influence on the septum bulge as well. This, however, is an artifact due to the measurement precision of the used instrument. For the two pressurized setups a steady increase of septum bulge over time is visible, while the amount by which it increases is enhanced by both factors, temperature/humidity and pressure as can be seen in Table 6. The unpressurized samples were conducted as controls. The highest measured septum bulge increase between months 1 and 12 was for a sample of the 1370 kPa under accelerated room temperature conditions. However, we do not expect it to be a problem as the bulge increase is much lower compared to other experiments which were unproblematic.

According to the ICH Q1A (R2) guideline a 6-month experiment under accelerated conditions is the minimum necessary. Yet the experiment was conducted for the double time until the last value was measured. Besides that, the bulged septum did not show any signs of damage. ICH guideline Q1E determines the extrapolation. It is stated that the change pattern in the existing data shall be approximated with a regression function with good fit. Curves of the septum bulge clearly have logarithmic shape in most experiments we conducted, especially due to the behavior in the first 14 days. But the pattern of months 1 to 12 often looks more linear then logarithmic, because they start after the initial phase of septum bulging. A logarithmic fit may thus underestimate the septum bulge in predictions. We therefore decided to fit the curves with linear regression as linear functions always have a stronger growth than logarithmic functions in the predicting area of the curve and when calculated from the same parameters. As explanation: when fitting a logarithmic curve with a linear function the fit function always intersects twice with the logarithmic function. Since the logarithmic function is rightbound and positive, the slope of a linear function must be higher than the logarithmic function and an extrapolation with the linear fit always yields a higher value compared to the logarithmic function for an x-axis value larger than the second intersection of the two curves. In order to rather overestimate than underestimate septum bulge and the fact that most curves have rather linear shape, we decided for this ‘worse case’ approximation. With this estimation, the largest increase of an average septum bulge will be the 1370 kPa sample for room temperature with ~ 0.27 mm (see Y24 value of ACC FRG in Table 6). As we have seen septum bulge changes of double amount in other experiments (e.g., the 500 days experiment) which did not turn out to be a problem, we do not expect a problem here either. We therefore conclude from our experimental data and its analysis that a shelf-life time of 2 years in room temperature or refrigerator will be feasible regarding the septum of the cartridge in the autoinjector device we proposed.

### Secondary pressure failure mode

The most important finding of the secondary pressure failure mode experiments is that the lowest pressure at which a 3 mL cartridge broke is 4922 kPa which is more than 3.5 times higher than the highest pressure (1370 kPa) used for 3 mL cartridges in the autoinjector. Regarding the 1.5 mL cartridges the difference was even higher with 9226 kPa being almost 6.5 times higher than the maximum used pressure of 1420 kPa.

Regarding the differences in the cartridges, CB1 and CA1 have very different average breaking points (11,139 ± 3896 kPa vs. 7899 ± 830 kPa) even when having the same plunger (PE3). Unfortunately, the sample set of cartridge B1 with Plunger E3 came out with a significant Shapiro-Wilk test and therefore is not considered to be normally distributed. If we would conduct the t-test nonetheless, cartridge A1 and plunger E3 would have yielded a significant difference which would support the impression the values give. To prove this assumption additional experiments would have to be made. The experiments with CB1 and PD3 have a mean value of 11,375 ± 2743 kPa, which is quite similar to the CB1 + PE3 and different to CA1 + PE3. Especially when looking at the standard deviations of the different sample sets. Standard deviation of Company A cartridges is about 10% of the mean value (CA1 + PE3: ~10.5%, CA2*+PD4*: ~9.7%), standard deviation of company B cartridges is around 30% of the mean value (CB1 + PD3: ~24.1%, CB1 + PE3: ~34.9%). In this sense it seems as if company B cartridges are more resistant to pressure than company A cartridges but also with larger variation. This is especially interesting as pharma grade type I glass cartridges from different manufacturers are all formed from glass pipes they obtain from Schott AG (Mainz, Germany) according to the cartridge’s respective specifications. Therefore, the process of forming cartridges must have a significant influence on the properties of the different cartridges.

### Drug product stability

The size exclusion chromatography of Adalimumab drug product determined a monomer concentration of more than 98% in all samples thus satisfying the requirement. Low amounts of high molecular weight (HMW) residues indicate that there are no agglomerates in the drug products. The requirement of less than 2% HMW is satisfied for all samples. Also, low molecular weight residues which could indicate debris can only be found in small amounts. The combined amount of mAb light chains and heavy chains is in all cases above 99% complying with the requirement of at least 95%. The amount of non-glycosylated heavy chains (NGHC) is low. Deglycosylation of the heavy chains is associated with significantly reduced bioactivity [29] and must therefore be avoided. The non-reducing electrophoresis shows IgG to be the main component of the drug product. Subvisible particles was analyzed according to USP 787 and all drug products, independent of pressure duration, comply with the regulation.

Regarding other biologic drugs we assume that other mAbs will exhibit a similar stability under pressure, as they all have a very similar structure. For our pressure experiments we choose explicitly a mAb because this class of biologics are highly sensitive due to their complex compositions. Therefore, we further assume that antibody fragments and other biologic drugs with similar or less complex structures will behave similarly under the pressure conditions we applied in our experiments. The literature on cold pressure denaturation we cited in the introduction shows that mesophilic organisms are viable up to 40 MPa [20] (the max. pressure used in the autoinjector concept is 1.42 MPa), which also gives a clear hint about the potential stability of proteins in the device. Regarding the catalytic activity of enzymes, it may depend on the enzyme. Decaneto et al. [30] studied an enzyme under pressure and found that generally enzymatic activity can be influenced by pressure for different reasons as small changes in the protein structure, hydration, influence on the rate limiting step and also changes of conformation and hydration of the ligand occur; however, we do not believe this to be an issue in our case as hydration and small conformational changes are subject to a change in environment energy levels and are likely reversible when the pressure changes back to normal, and the drug product will be used under normal pressure levels in the body. Regarding RNA and DNA single strands are covalently bonded and should not be affected by pressure. Double stranded DNA molecules are likely unaffected at used pressures [31].

### Scalability and manufacturing of a new autoinjector concept

In order to make a new autoinjector concept successful on the market, we consider three main prerequisites. A new autoinjector device must outperform the state of the art with respect to human factors compliance (1) and technical versatility regarding the formulation to be delivered (2), all while its cost must be in scope of devices on the market (3).

Autoinjectors are commonly manufactured in mass production; therefore, the use of standardized primary packaging materials suitable for standard filling lines is of particular importance and was the leitmotif for the choice of components in the tests of this article and the development of the device. Also, the assembly of the autoinjector device from its subassemblies and merging the filled primary container for the final combination product is subject to this necessity, which has been successfully assessed, but exceeds the scope of this article.

## Conclusion

The goal of the test program was to check whether pre-pressurized cartridges lead to problems when used in the given autoinjector concept. Not all primary packaging materials are suitable (e.g., bilayer septa), but there are existing and established standard primary packaging materials that proved to satisfy the requirements with good tolerance. Especially the septum bulge tests under accelerated storage conditions give important evidence on the ability of the primary packaging to withstand the pressure for the full shelf-life of a medicine. For the glass stability it was especially interesting to see how much glass cartridges from different manufacturers differ, despite being crafted from the same raw material. However, since all breaking points independent of the cartridge type were magnitudes above the pressures used in our proposed autoinjector device, we do not see an issue for usage, independent of the manufacturer, as long as maximum pressures are not exceeded. Regarding the Adalimumab drug product, we do not see any signs of decay in the release testing. This was expected with regard to the literature on protein denaturation under pressure; but the tests were nonetheless necessary as proteins in their formulation are not only subject to denaturation, but also to aggregation and agglomeration which must not happen. It was therefore especially important to us to show with a release testing in a GMP setting that the drug product is not altered.

From the results we conclude that regarding primary packaging and drug product all prerequisites for the technology of the autoinjector can be met. Thus, it is possible to develop an autoinjector device which is by default in the extrusion phase and bypasses the transient phase described by Veilleux et al. [9]. Since glass and many plastics are of inelastic nature, deformation and breakage or jamming of devices in the transient phase is possible. It can therefore be especially interesting to be able to avoid larger pressure spikes in a device as it is for the concept used in this publication.

Any final medicinal product will have to be tested for stability for the whole shelf-life, therefore a further test in the final device will be conducted once a combination product is brought to the market. As for the primary packaging and the drug product we consider all general critical issues to be assessed and diverted.

## Data Availability

The datasets generated during and/or analysed during the current study are not publicly available due to company policy but are available from the corresponding author on reasonable request.

## Abbreviations

MV: Mean value
SD: Standard deviation
GF: Glide force
BLF: Break-loose force
mAb: monoclonal antibody
CH: Cartridge holder
SEC: Size Exclusion Chromatography
CE R/NR: Capillary electrophoresis reducing/non-reducing
USP/EP: United States Pharmacopeia / European Pharmacopeia
ISO: International Organization for Standardization
GMP: Good Manufacturing Practice
PFS: Pre-filled syringe
ICH: International conference on harmonization of technical requirements for registration of pharmaceuticals for human use
